# Corrigendum

**DOI:** 10.1111/bpa.13127

**Published:** 2022-10-28

In this paper [**1**], Cancer Research UK should be removed from Funding Information.

The online version has been corrected.
